# Platelet Responses to Urethane Dimethacrylate-Based Bone Cements Containing Monocalcium Phosphate/ε-Polylysine: Role of ε-Polylysine in *In Vitro* Wound Healing Induced by Platelet-Derived Growth Factor-BB

**DOI:** 10.1021/acsmaterialsau.4c00143

**Published:** 2025-01-03

**Authors:** Phatchanat Klaihmon, Piyarat Sungkhaphan, Boonlom Thavornyutikarn, Setthawut Kitpakornsanti, Praphasri Septham, Anne Young, Chanchao Lorthongpanich, Wanida Janvikul, Weerachai Singhatanadgit

**Affiliations:** † Siriraj Center of Excellence for Stem Cell Research, Faculty of Medicine Siriraj Hospital, Mahidol University, Bangkok 10700, Thailand; ‡ National Metal and Materials Technology Center, National Science and Technology Development Agency, Pathum-thani 12120, Thailand; § Faculty of Dentistry and Research Unit in Mineralized Tissue Reconstruction, Thammasat University (Rangsit Campus), Pathum-thani 12121, Thailand; ∥ Division of Biomaterials & Tissue Engineering, UCL Eastman Dental Institute, London NW3 2PF, U.K.; ⊥ Blood Products and Cellular Immunotherapy Research Group, Faculty of Medicine Siriraj Hospital, Mahidol University, Bangkok 10700, Thailand

**Keywords:** platelet activation, monocalcium phosphate, polylysine, urethane dimethacrylate, poly(methyl methacrylate), platelet-derived growth factor

## Abstract

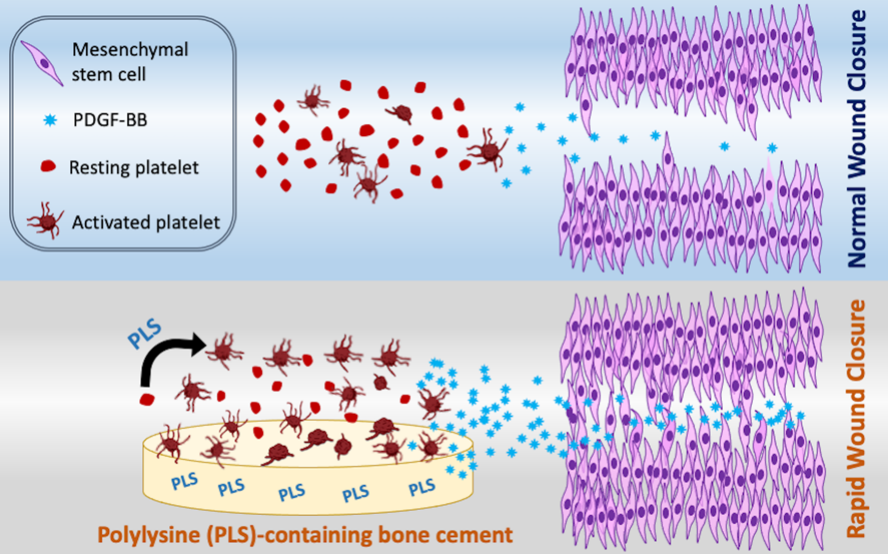

Platelets play a pivotal role in initiating bone fracture healing. However, the interaction between platelets and bone cements used for fracture repair remains relatively unexplored. This study investigated the platelet response to recently developed urethane dimethacrylate-based bone cements containing 8% (w/w) monocalcium phosphate monohydrate (MCPM) and/or 5% (w/w) ε-polylysine (PLS). All experimental bone cements achieved final monomer conversions of 75–78%, compared with the 86% conversion of the commercial PMMA bone cement Kyphon. The MCPM and PLS microparticles, varying in size, were dispersed within the glass-filler-incorporated polymer matrix. In contrast to Kyphon, all experimental cements exhibited significantly smoother and more hydrophilic surfaces. Bone cements incorporating PLS, with or without MCPM, effectively activated platelets by inducing cellular adhesion, aggregation, and extracellular-signal-regulated kinase (ERK) activation, comparable to Kyphon. Flow cytometry analysis demonstrated a statistically significant increase in CD62P-positive platelets following exposure to PLS-incorporated bone cements and exogenously administered PLS in a concentration-dependent manner, but not with Kyphon. A wound healing assay revealed a 2-fold enhancement in wound closure within 24 h and exceeding 85% at 48 h by bone cements containing PLS, with or without MCPM, and Kyphon. Notably, platelet-derived growth factor BB (PDGF-BB) secretion was significantly elevated, specifically after platelet exposure to PLS-incorporated bone cements, a phenomenon not observed with Kyphon. Interestingly, PDGF-BB neutralization attenuated wound closure induced by the PLS-incorporated bone cements. In conclusion, the urethane dimethacrylate-based bone cements containing PLS demonstrated a significant enhancement in platelet activation and PDGF-BB secretion, which, at least partly, enhanced *in vitro* wound closure. The results suggest that PDGF-BB plays a crucial role in the PLS-mediated enhancement of wound healing in these bone cements.

## Introduction

Following a bone fracture, the body initiates a complex cascade of events to heal the fracture and restore functionality. This multiphase process includes hemostasis, inflammation, repair, and remodeling. Fracture repair is a complex biological process that can be significantly aided by surgical interventions and the use of bone cements. Bone cement plays a crucial role in this procedure by providing fixation and support. Poly(methyl methacrylate) (PMMA) has emerged as the most preeminent and extensively utilized bone cement across a diverse array of medical applications, including orthopedic and craniomaxillofacial surgery, as well as traumatology.

In pursuit of enhanced therapeutic outcomes in fracture healing facilitated by PMMA, novel bone cements boasting superior characteristics have emerged. These advancements have been achieved through the incorporation of high molecular weight dimethacrylate monomers, such as urethane dimethacrylate (UDMA) – a material prevalent in dental composites,^1^ and oligomers, such as poly(propylene glycol) dimethacrylate (PPGDMA). The strategic utilization of these components aimed to optimize the polymerization and mechanical properties as well as the cytocompatibility of the cement,^1−4^ potentially leading to improved clinical outcomes.

UDMA-based bone cements, incorporated with a bioactive calcium phosphate, i.e., monocalcium phosphate monohydrate (MCPM), and a cationic preservative, *i.e.*, ε-polylysine (PLS), have recently been formulated with suitable physical, chemical and mechanical properties for the use in fracture repair.^5^ However, studies on their biological responses have been limited to certain bone cells and inflammatory cells. It has been shown that UDMA-based bone composites, containing both MCPM and HA, promoted the growth of mesenchymal stem cells (MSCs), leading to subsequent mineralization while inhibiting the proliferation of fibroblasts, osteoclast progenitors, and peripheral blood mononuclear cells (PBMCs).^6^

Following the implantation of biomaterials, including bone cements, vascular injury triggers an immediate hemostatic response characterized by vasoconstriction of the damaged vessel and platelet activation. Within minutes after blood vessel injury, a fibrin clot forms at the site of injury.^7^ The initial hemostatic phase is followed by the inflammatory response, the proliferative phase characterized by robust tissue regeneration, and ultimately the remodeling phase for functional restoration.^8^ Thus, platelets play a pivotal role in initiating the wound-healing cascade, acting as the first cellular responders at the site of injury.^7^ Beyond their well-established role in hemostasis, a growing body of experimental and clinical data implicates platelets as significant modulators in various physiopathological processes, including wound healing and bone repair.^9−11^ This expanded functionality is attributed to their release of growth factors, cytokines, and extracellular matrix (ECM) modulators. Together, these act in a coordinated manner to promote the migration, proliferation and differentiation of mesenchymal stem cells (MSCs) toward tissue-specific lineages,^9^ including osteogenic lineage for bone repair.

Upon exposure to inductive stimuli, platelets undergo activation. Akt-dependent signalings play an important role in platelet activation. Animal studies showed that platelets lacking Akt have defects in their functions, including aggregation, fibrinogen binding, and granule secretion.^12,13^ Studies using Akt inhibitors in human platelets generally also support a similar role for Akt in activating human platelets.^14−16^ In addition, within platelets, Akt-independent signaling pathways, such as the two mitogen-activated protein kinases (MAPKs), i.e., ERK and p38MAPK, have been implicated in signaling pathways.^17−20^ Upon activation, platelets rapidly translocate large quantities of a cell surface adhesion molecule CD62P (P-selectin) from their intracellular α-granules to their cell surface.^21,22^ These molecules facilitate interaction with surrounding cells and fibrin, alongside morphological changes and the activation of intracellular signaling pathways.^10^

Upon activation, platelets degranulate and release a repertoire of well-characterized soluble mediators that have been implicated in promoting the healing of injured tissues, such as bone fractures. Mediators, include platelet-derived growth factor-BB (PDGF-BB), transforming growth factor-β1 (TGF-β1), platelet factor 4 (PF-4), and stromal cell-derived factor 1 alpha (SDF-1α).^23^ PDGF-BB, one of the first growth factors to be released into the wound environment by platelet degranulation, is also secreted by monocyte-derived cells, fibroblasts, and endothelial cells in later stages of wound healing.^24^ It thus plays an important role in multiple subsequent stages of wound healing and tissue repair. Its role includes stimulation of MSC proliferation and migration and promoting the formation of granulation tissue, ECM production, and angiogenesis.^24^ TGF-β1, secreted in high amounts by activated platelets, acts as a multifaceted regulator in wound healing and influences various processes, including modulation of inflammation, fibroblast activation and collagen synthesis, ECM remodeling, and wound contraction.^23,25^ PF4 exerts chemotactic activity, recruiting monocytes and neutrophils to the wound site.^26^ SDF-1 is a crucial chemokine, directing the homing and migration of stem and progenitor cells, and mediates migration, proliferation and function of endothelial progenitor cells.^27^ Collectively, these factors orchestrate the recruitment and activation of many cell types, including mesenchymal stem cells (MSCs), at the site of injury, consequently promoting tissue repair and regeneration.^28^

As platelets are the first cells to respond to tissue injury, initiating the wound healing cascade at the site of damage, investigating their responses to implanted bone cements is crucial. These responses may significantly influence the success of fracture repair. Therefore, this study aimed to investigate the platelet responses to the UDMA-based bone cement pastes incorporated with MCPM and PLS. Additionally, the functional consequences of the bone-cement-induced platelet activation were evaluated using an *in vitro* MSC-based wound healing assay.

## Materials and Methods

### Materials

A commercial PMMA (Kyphon HV-R; high viscosity PMMA bone cement with 30% barium sulfate) was obtained from Medtronic, USA. All chemicals used for composite preparation were of analytical grade: urethane dimethacrylate (UDMA, a base monomer) (Rahn AG, Switzerland, MW = 470), poly(propylene glycol) dimethacrylate (PPGDMA, a diluent monomer) (Polysciences Inc., USA, MW = 560), hydroxyethyl methacrylate (HEMA, a diluent monomer) (Sigma-Aldrich, St. Louis, USA), benzoyl peroxide (BPO, an initiator) (Sigma-Aldrich), N-tolyglycine glycidyl methacrylate (NTGGMA, an activator) (Esstech Inc., USA), monocalcium phosphate monohydrate (MCPM, a bioactive filler, average particle size = 53 μm) (Himed, Old Bethpage, USA), ε-polylysine (PLS) (particle size = 20–40 μm, Handary, Brussel, Belgium), and silane treated aluminosilicate glass particles (fillers, average diameters = 0.7 and 7 μm) (DMG, Hamburg, Germany).

### Bone Cement Preparation

#### Bone Cement Paste Preparation

UDMA-based composites (CPs) were formulated by combining initiator and activator pastes. Each paste consisted of a liquid phase (i.e., monomers with initiator or activator) (Table S1) and a powder phase (i.e., glass + MCPM + PLS). For the liquid phases, UDMA and PPGDMA were mixed in a fixed weight ratio of 2:1, followed by the addition of HEMA at 2.5 wt %. After homogeneously mixing, BPO (1.5 wt %) was incorporated to generate the initiator liquid, while NTGGMA (1 wt %) was added to form the activator liquid. Both liquid phases were stirred at room temperature for 2 h and subsequently sonicated at 40 °C for 2 h to ensure complete dissolution of the solid particles.

To produce the pastes, aluminosilicate glass particles (average diameters of 0.7 and 7 μm), combined in a 1:1 weight ratio, were integrated into both initiator and activator liquids. MCPM (8 wt % of the total CP) was solely integrated into the initiator paste, whereas PLS (5 wt % of the total CP) was equally added into both initiator and activator pastes (Table 1). The specific formulation containing 8% MCPM and 5% PLS (8M5P) has previously been tested and found to have handling/setting characteristics and mechanical properties suitable for bone cement requirements.^29,30^ The control cement composite formulated without MCPM and PLS was also simultaneously prepared. The ratio of total powder to liquid phases was maintained at 3:1 in all cement pastes. Thorough mixing of the liquid and powder phases was achieved using an integrated mixer and a deaerator system (Mazerustar, KK-250S, Kurabo, Japan) at a rotation speed of 4300 rpm for 8 min.

**Table 1:** Amounts of MCPM and PLS in Each Bone Cement Formulation Tested in the Present Study

	wt % (of total composite paste)	
Formulations	MCPM	PLS
Control composite	0	0
8M	8	0
5P	0	5
8M5P	8	5

#### Bone Cement Disc Preparation

To prepare composite discs, the initiator and activator pastes for the individual formulated cements were combined in a 1:1 weight ratio on a paper surface and thoroughly mixed for 1 min. Then, the resulting mixtures were separately transferred to metal molds with a 10 mm inner diameter and a 1 mm thickness. The entire specimens were placed in an oven and maintained at 37 °C for 24 h to allow for complete curing. After the curing process, the composite discs were carefully removed from the molds, and any excess overflow material was removed. A commercial PMMA cement (Kyphon HV-R, coded as Kyphon), mixed as per the manufacturer’s instructions within their use-by date, was used as a comparison. The prepared discs were UV sterilized before being used in cell studies.

### Monomer Conversion

The degree of (monomer) conversion of the composites and Kyphon at 40 min postmixing was determined by Attenuated Total Reflection-Fourier Transform Infrared spectroscopy (ATR-FTIR) (THERMO/Nicolet 6700 FTIR spectrometer) at 25 °C, as previously described.^6^ The monomer conversion (MC) at a given time (*t*) after mixing was calculated from the peak heights at 1636 cm^–1^ determined at the initial time (*H*_i_) and after time *t* (*H*_*t*_) above the background using the following equation:
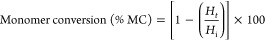
1

### Elemental Composition and Particle Distribution

To analyze the elemental composition and spatial distribution within the formulated bone cement, a scanning electron microscope equipped with energy-dispersive X-ray analysis (SEM/EDX-EBSD, HITACHI/S-3400N, Japan) was utilized. The cross-sectional surface of the fractured bone cement disc specimen was examined under an accelerating voltage of 20 kV. Each surface square was mapped over a 200-s acquisition time, with a 6 min count rate optimization.

Due to the limitations of EDX in detecting light elements such as nitrogen, electron probe microanalysis (EPMA) was also employed to corroborate the EDX results. Prior to EPMA analysis, the cross-sectional surface of the specimen disc was coated with carbon using the Quorum Q150EE plus model for 60 s. Elemental mappings for calcium (Ca) and nitrogen (N) in the bone cement were then performed using an EMPA analyzer (Shimadzu model 8050G). Additionally, the atomic weight percentages of both organic and inorganic elements were determined.

### Surface Characterization

The surface topography, roughness, and hydrophilicity of the developed bone cements were characterized by SEM, atomic force microscopy (AFM), and water contact angle analysis, respectively. The SEM samples were primarily sputter-coated with gold and subsequently observed using a JEOL JCM 6000 scanning electron microscope (JEOL UK, Welwyn Garden City, UK) under an accelerating voltage of 10 kV.

AFM surface topography images of all the cement discs investigated were obtained in ambient air using Nanosurf’s CoreAFM (Nanosurf AG, Switzerland) operating in a phase contrast mode with a vibration frequency of 166 kHz, vibration amplitude of 470 mV, 256 scanning lines/scan, and a measuring speed of 9 s/line. The scan size was set at 20 μm × 20 μm. A Nanosurf CoreAFM control program version 3.10.0.23 was used to analyze the resulting images. The surface roughness (Sa) values (nm) in four random fields per sample were obtained.

Water contact angles observed on the bone cement discs were measured using a contact angle measurement and drop shape analysis system (OCA 25) with SCA20 analysis software version 6.1.19 build 6019 (Dataphysics, Germany). A 2 μL drop of distilled water was placed onto each bone cement disc to obtain a static contact angle. Four different substrate fields were measured per sample. The static water contact angles were employed to compare the wetting characteristics of the different bone cement surfaces.^31^

### Isolation of Platelet-Rich Plasma (PRP)

To obtain single-donor PRP samples, blood samples were taken from healthy donors who had not taken any drugs during the 10 days before sampling. Twenty milliliters of blood were collected into a citrate-anticoagulant tube, and the PRP was prepared by centrifuging the blood at 500g for 5 min and 2000g for 5 min at room temperature. The PRP was immediately isolated after the centrifugation step and kept on ice for subsequent uses. This study was ethically approved by the Ethics Review Sub-Committee for Research Involving Human Research Subjects of Thammasat University No. 3 (COA No. 068/2564), the Institutional Biosafety Committee of Thammasat University (057/2564) and the Institutional Review Board of the Faculty of Medicine Siriraj Hospital (COA. No. 733/2557 (EC1), Si101/2015).

### Exposure of Bone Cements to PRP

One milliliter of PRP was added to each bone cement disc in a 24-well plate (Corning), and the whole plate was incubated in a 37 °C humidified incubator on a 3D-sunflower mini-shaker (Biosan) for indicated times.

### SEM of Platelet Adhesion and Aggregation

After exposure to PRP for 30 min, the bone cement samples were washed with phosphate-buffered saline (PBS) and fixed in 100 μL of 3% glutaraldehyde for 2 h, followed by washing with distilled water. The samples were dehydrated with increasing concentrations of ethanol, dried in an incubator at 60 °C, and subsequently sputter-coated with gold using a gold sputter coater. The gold-sputtered platelet layers on the bone cement samples were examined with a JEOL JCM 6000 scanning electron microscope (JEOL UK, Welwyn Garden City, UK) under an accelerating voltage of 10 kV.

### Immunofluorescence Staining

To demonstrate bone-cement-induced platelet aggregation, the materials were exposed to PRP for 30 min, fixed in 4% paraformaldehyde, washed, and blocked with 5% bovine serum albumin in PBS. Then, Alexa Fluor 568-conjugated phalloidin (dilution at 1:500; Invitrogen) was added to stain the platelet cytoskeleton (F-actin). Immunofluorescence images were taken under a confocal fluorescence microscope (Nikon Ti Eclipse, Nikon Instruments Inc., NY, USA).

### Analysis of Platelet Activation by Flow Cytometry (FCM)

Samples of 10 μL of bone-cement-exposed PRP were sampled at indicated times (30 and 60 min) and diluted with 90 μL PBS in a 12 × 75 mm fluorescence-activated cell sorting (FACS) polystyrene tube. Two microliters each of FITC-conjugated anti-human CD42b and PE-conjugated anti-human CD62P antibodies (Biolegends) were added, and stained samples were incubated at room temperature for 15 min in the dark. Afterward, 300 μL of 1% paraformaldehyde in PBS was added before being subjected to FCM analysis by FACS Canto flow cytometer (BD Biosciences). CD42b^+^CD62P^+^ cells were considered activated platelets.

To assess the role of PLS and MCPM in platelet activation, PRP samples were treated with exogenously added PLS (100, 200, and 600 μg/mL) and MCPM (5, 15, and 30 μg/mL) for 30 and 60 min. The samples were then stained and analyzed as described above.

### Western Blotting Analysis

Bone-cement-exposed PRP samples were collected at 5 min intervals for a period of 20 min following exposure, resuspended in Tyrode’s solution (Sigma-Aldrich), and centrifuged at 3000 rpm for 5 min to pellet the platelets. Protein lysates from platelet pellets were extracted using RIPA lysis buffer (Cell Signaling Technology), and protein concentration was quantified by BCA Protein Assay Kit (Thermo Fisher Scientific). Equal amounts of protein lysate were dissolved in 10% polyacrylamide gel and transferred onto the PVDF membrane. Membranes were blocked with 5% skim milk/TBST buffer and incubated with anti-total Akt, anti-phosphorylated Akt (p-Akt) (Ser473), anti-total ERK, anti-phosphorylated ERK (p-ERK), and anti-β-actin antibodies (1:1000, Cell Signaling Technology) overnight at 4 °C. Membranes were then washed and incubated with corresponding HRP-conjugated secondary antibodies for 1 h at room temperature. Immune complexes were visualized by enhanced chemiluminescence. Chemiluminescent images were taken using Image Quant LAS400 software. The expression of β-actin was used as an internal control. The activation of Akt and ERK signaling pathways in platelets was determined by quantifying the expression of phosphorylated Akt (p-Akt) and phosphorylated ERK (p-ERK). Band intensities of each bone cement sample were normalized to that of the untreated samples at similar time points (set to 1.0 in the untreated group). The p-Akt/Akt and p-ERK/ERK ratios (vs untreated samples at the same time points) were analyzed.

### Enzyme-Linked Immunosorbent Assay (ELISA)

After 60 min exposure to bone cements, the PRP samples were collected and centrifuged at 3000 rpm for 5 min to pellet the platelets. The resulting platelet-free bone-cement-exposed plasma supernatants were collected and subsequently used to quantify cytokines and chemokines, including PDGF-BB, PF4, TGF-β, and SDF-1α, using ELISA kits (R&D systems, Abingdon, UK) according to the manufacturer’s instructions.

### Scratch-Based Wound Healing Assay

Bone marrow-derived MSCs purchased from the American Type Culture Collection (ATCC, USA) were plated onto a 24-well plate in a low-glucose DMEM medium containing penicillin/streptomycin and 10% fetal bovine serum (FBS) overnight. The one-milliliter tip was used to create a wound on the MSC culture. The old culture medium was replenished with a low-glucose DMEM medium containing penicillin/streptomycin and 10% platelet-free bone-cement-exposed plasma samples. MSC culture media with 10% FBS and without FBS were used as controls. Wound closure photomicrographs were taken at 24-h intervals for a period of 48 h.

### PDGF-BB Neutralization Assay

To ascertain the specific role of PDGF-BB in the wound-healing acceleration mediated by bone-cement-exposed PRP, a PDGF-BB neutralization assay was employed. The neutralizing antibody against human PDGF-BB (#AF-220-NA, R&D systems) or an isotype control antibody (final concentration at 500 ng/mL) was added to pretreat the bone-cement-exposed PRP for 1 h before being used to supplement the culture medium for the scratch-based wound healing assay.

### Data and Statistical Analyses

FlowJo software (Version 10.0.0) was used to analyze flow cytometric data, while ImageJ software (Version 1.53) was used to determine wound closure space. All data were presented as mean ± SD, and statistical analysis was performed using one-way analysis of variance (ANOVA) with posthoc Dunnett’s test via SPSS software (SPSS, Inc., Chicago, USA). A threshold of *p*-value <0.05 was considered statistically significant.

## Results

Upon polymerization after the thorough mixing of the initiator and activator pastes, C = C bonds in the (di)methacrylate (macro)monomers were converted to C–C bonds through a rapid free radical chain reaction. The monomer conversions were analyzed by ATR-FTIR, as described in the Materials and Methods, and found to be 75%, 76%, 77%, 78%, and 86% for control composite, 5P, 8M, 8M5P, and Kyphon samples, respectively.

### Particle Distribution and Bulk Elemental Composition

**Figure 1:**
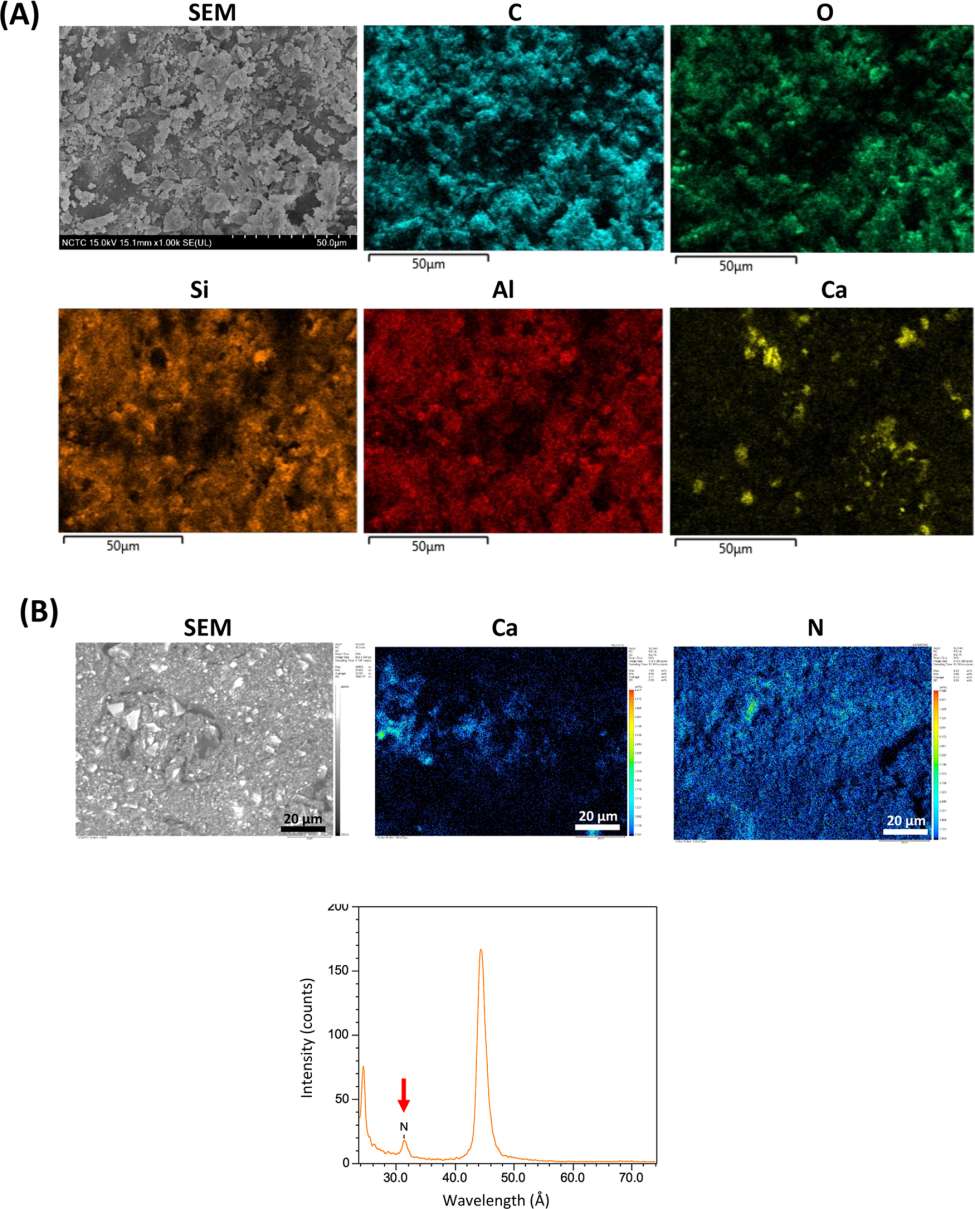
SEM images and corresponding elemental mappings of the cross-sectional surfaces of the 8M5P specimens analyzed by EDX (A) and EPMA (B). Notably, while nitrogen (N) was not detectable by EDX, it was successfully identified by EPMA, with a peak wavelength of 31.3 Å (red arrow).

To observe the distributions of both PLS and MCPM particles in the polymeric matrix of the bone cement, the cross-sectional surfaces of 8M5P discs were examined using both SEM/EDX and EPMA analyses. Figure 1A presents SEM/EDX elemental mappings for carbon (C), oxygen (O), silicon (Si), aluminum (Al), and calcium (Ca), and Figure 1B shows EPMA elemental mappings for Ca and nitrogen (N). The spatial distribution of the polymer phase was visualized through the element maps for C and O, the primary constituents of the monomers. The homogeneous distributions of O, Si, and Al, derived from the glass fillers (0.7 and 7 μm), were explicitly observed within the polymer matrix. In Figure 1B, the MCPM microparticles were randomly detected in varied sizes, as seen in the calcium maps taken by both SEM/EDX and EPMA. The PLS microparticles were, however, solely observed by EPMA due to the difficulty in light element detection by EDX in the less prominent regions in the nitrogen map, due to its relatively smaller quantity incorporated, and detected at the peak wavelength of 31.3 Å (pointed with a red arrow in Figure 1B).

The cross-sectional elemental composition of the 8M5P bone cement specimen, analyzed by EPMA, is presented in Table 2. By weight, carbon and oxygen were the predominant elements (80.2%), followed by silicon and aluminum from the glass fillers, which were present at approximately 15%. MCPM was detected with a calcium-to-phosphorus ratio of 1:2.5, and nitrogen-containing PLS were also clearly evident within the bulk of the 8M5P bone cement. It is noteworthy that the rather uniform dispersion of all individual particles observed in the 8M5P formulation provided evidence that the preparation procedure was sufficiently effective for all formulations.

**Table 2:** Cross-Sectional Elemental Composition of the 8M5P Bone Cement Specimen, Analyzed by EPMA

Elements	Weight (%)
C	41.6
O	38.6
Al	1.8
Si	13.3
Ca	0.4
P	1
N	3.3

### Surface Properties of Bone Cements

**Figure 2:**
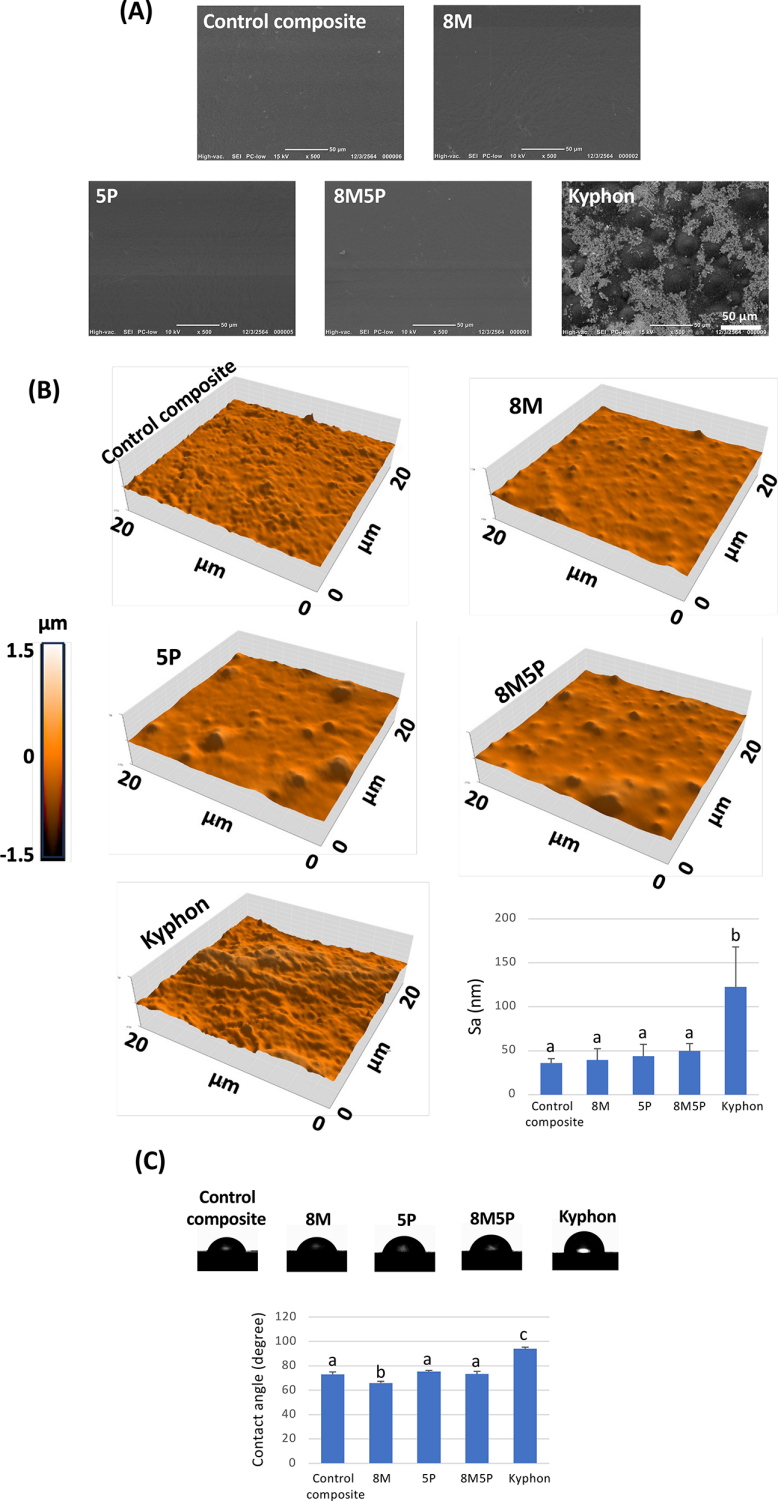
Surface characterization of the prepared bone cements. (A) SEM micrographs of surface characteristics of bone cements. (B) AFM images of surface characteristics of bone cements and a summary of surface roughness values. (C) Water drop shape images observed on bone cement surfaces and a summary of water contact angles. Data are expressed as mean ± SD (*n* = 4). In bar graphs, values signed with the same letter indicate that there was no significant difference between them.

Low-magnification SEM images of surfaces of all prepared bone cements are shown in Figure 2A. The surface morphologies of all 4 UDMA-based bone cements appeared smooth and homogeneous, whereas that of Kyphon revealed polymeric spheres with a variable diameter between 10 and 50 μm surrounded with barium sulfate agglomerates. The higher magnification AFM images of the bone cement surfaces further demonstrated the nano- to microroughed surface topography of the UDMA-based composites and the relatively more irregular and rougher surface of the Kyphon specimen. The average roughness (Sa) values of the UDMA-based composites and Kyphon were 39–50 and 123 nm, respectively. The water contact angles measured on the individual bone cement surfaces are summarized in Figure 2C. Among the experimental bone cements, 8M possessed the highest surface hydrophilicity, according to its lowest water contact angle (65 ± 1.4°). The water contact angles of the control, 5P and 8M5P cement samples were 73 ± 1.9°, 75 ± 0.9° and 73 ± 2.2°, respectively, indicating that the addition of MCPM, but not PLS, helped enhance the wettability of the composite cement; MCPM appeared more slightly hydrophilic than the silane treated aluminosilicate glass. Despite its highest surface roughness, the water contact angle observed on the Kyphon surface was rather high (94 ± 1.3°), which was significantly greater than those of the experimental UDMA-based cements.

### Effects of Bone Cements on Platelet Adhesion and Aggregation and the Activation of Akt and ERK Signaling Pathway

**Figure 3:**
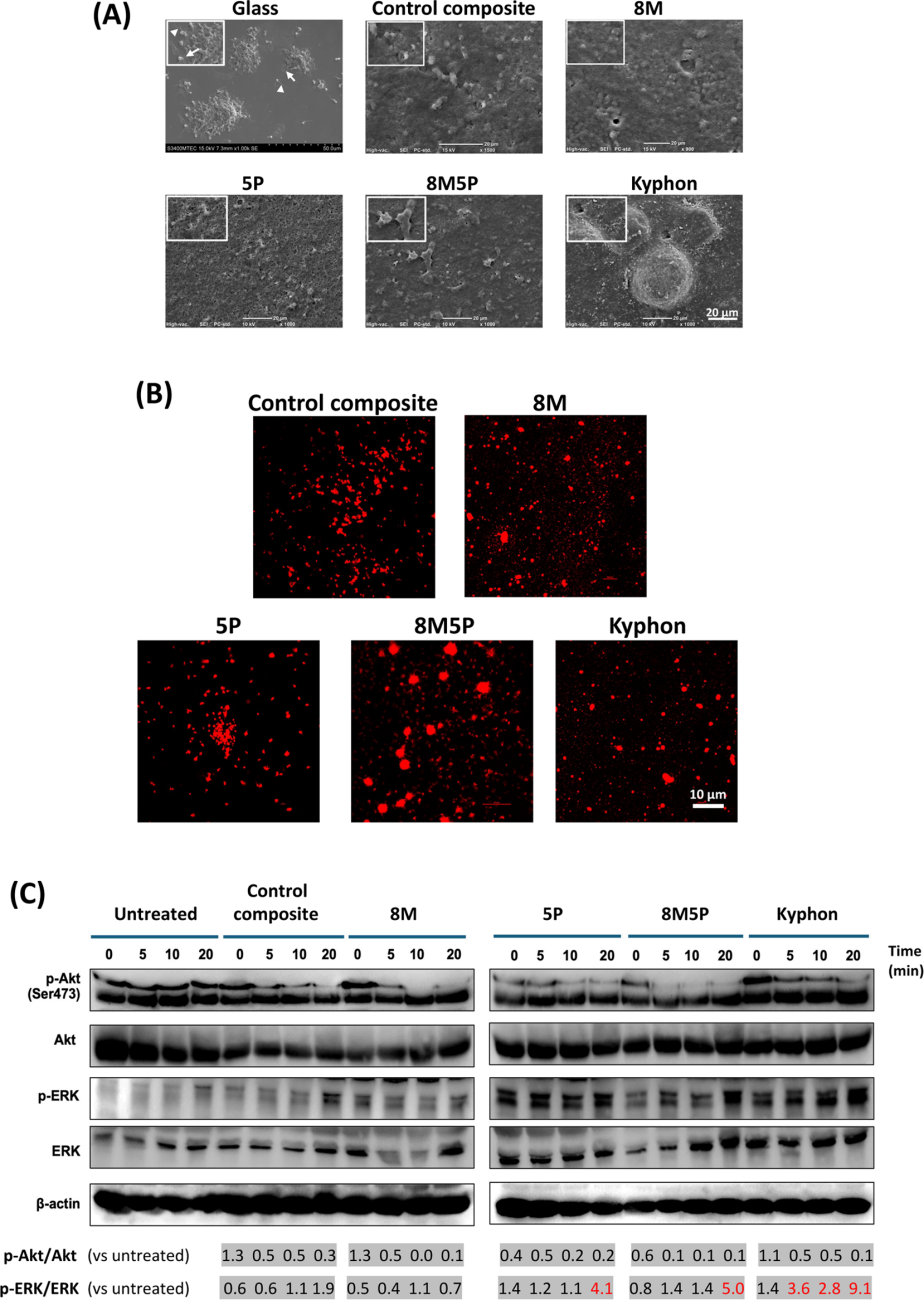
Effect of bone cements on the platelet aggregation and the activation of Akt and ERK pathways. Human PRP was cultured on the different bone cements for 30 min, and bone-cement-induced changes of platelets were visualized using SEM (A) and confocal fluorescence microscopy (B). Insets in (A) show a closer view of the platelet and surrounding matrix. In (C), the activation of Akt and ERK signaling pathways in platelets cultured on the bone cements was assessed at 5 min intervals for a period of 20 min following exposure, by quantifying the expression of phosphorylated Akt (p-Akt) and phosphorylated ERK (p-ERK) using Western blot analysis. The expression of β-actin was used as an internal control. The p-Akt/Akt and p-ERK/ERK ratios (vs untreated samples at the same time points) are shown below the Western blot bands. Band intensities of each bone cement sample were normalized to that of the untreated samples at similar time points (set to 1.0 in the untreated group). Ratios in red indicate increases more than 2.0-fold. All results shown are representative from independent experiments using 2 different single-donor PRP samples.

Representative micrographs in Figures 3A and 3B demonstrate platelet adhesion and aggregation on the different bone cement surfaces, visualized by SEM and confocal fluorescence microscopy. Figure 3A reveals the differential ultrastructural features of platelets adhering and aggregating on the tested bone cement surfaces compared with that of the control glass surface. On the control surface, well-adhered and spread platelets displayed extensive filopodia (arrowhead), visible as small branches protruding from the cell body (2–3 μm in diameter), and membranous lamellipodia (arrow). Well-aggregated platelets in groups ranging from small to as large as 50 μm were also observed, indicating activation. All tested bone cements appeared to alter platelet morphology. Notably, the 5P bone cement was covered by a platelet-embedded loose fibrinous structure while other cements displayed platelet-embedded dense membranous structures completely covering their surfaces. Clear extensive filopodia and lamellipodia with well-defined platelet margins were less frequently observed on all experimental bone cements after 30 min incubation compared with those seen on the glass surface. Figure 3B, with representative confocal fluorescence images of actin staining, further supports the presence of platelet adhesion and aggregation on all tested bone cements. Notably, the 8M5P bone cement appeared most effective in inducing platelet adhesion and aggregation. Compared with that of the untreated sample (no exposure to any bone cement at all time points studied), phosphorylation of Akt in platelets seemed to be markedly reduced by all the bone cements after 5–20 min of exposure (Figure 3C). In contrast, the 5P, 8M5P and Kyphon bone cements induced phosphorylated ERK in platelets for approximately 4, 5, and 9 folds after at least 20 min postexposure.

Taken together, the 5P and 8M5P formulations and the commercial PMMA Kyphon seemed to effectively, but not differentially, activate platelets by inducing cellular adhesion, aggregation and ERK activation.

### Effects of Bone Cements on Platelet Activation

**Figure 4:**
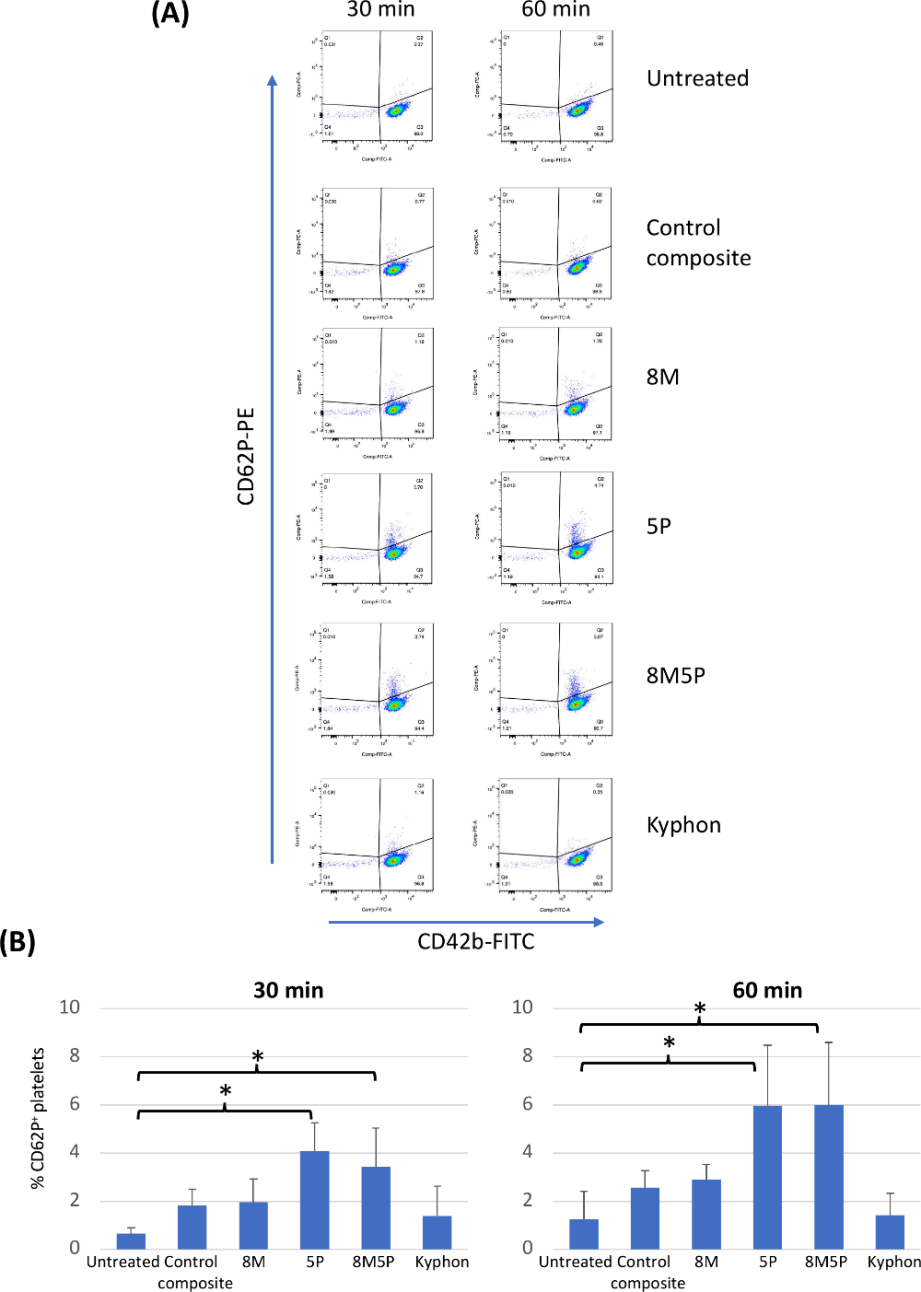
Effect of bone cements on the platelet activation assessed by FCM. Human single-donor PRP (from 4 donors) was cultured on the different bone cements for 30 and 60 min, and the cells were stained for CD42b and CD62P before FCM analysis. The gating strategy to measure the proportion of activated platelets (CD42b^+^CD62P^+^) is shown in (A), and a summary of % CD62P^+^ platelets induced by the different bone cements is shown in (B). Data are expressed as mean ± SD from three different single-donor PRP samples. Plastic surfaces were used for untreated samples. *p* < 0.05.

The gating strategy of CD62P-expressing CD42b^+^ platelets (activated platelets) is illustrated in Figure 4A. Representative FCM dot plots in Figure 4A demonstrate that compared with that of the control, only the experimental 8M, 5P, and 8M5P bone composites increased the number of platelets (CD42b^+^ cells) that expressed the platelet activation marker CD62P after 30 and 60 min of exposure. Consistent with this observation, analysis of the results revealed a statistically significant increase in the number of CD62P^+^ platelets by the 5P and 8M5P bone composites, but not by the 8M group, at both time points studied (Figure 4B).

### Effects of PLS and MCPM on Platelet Activation

**Figure 5:**
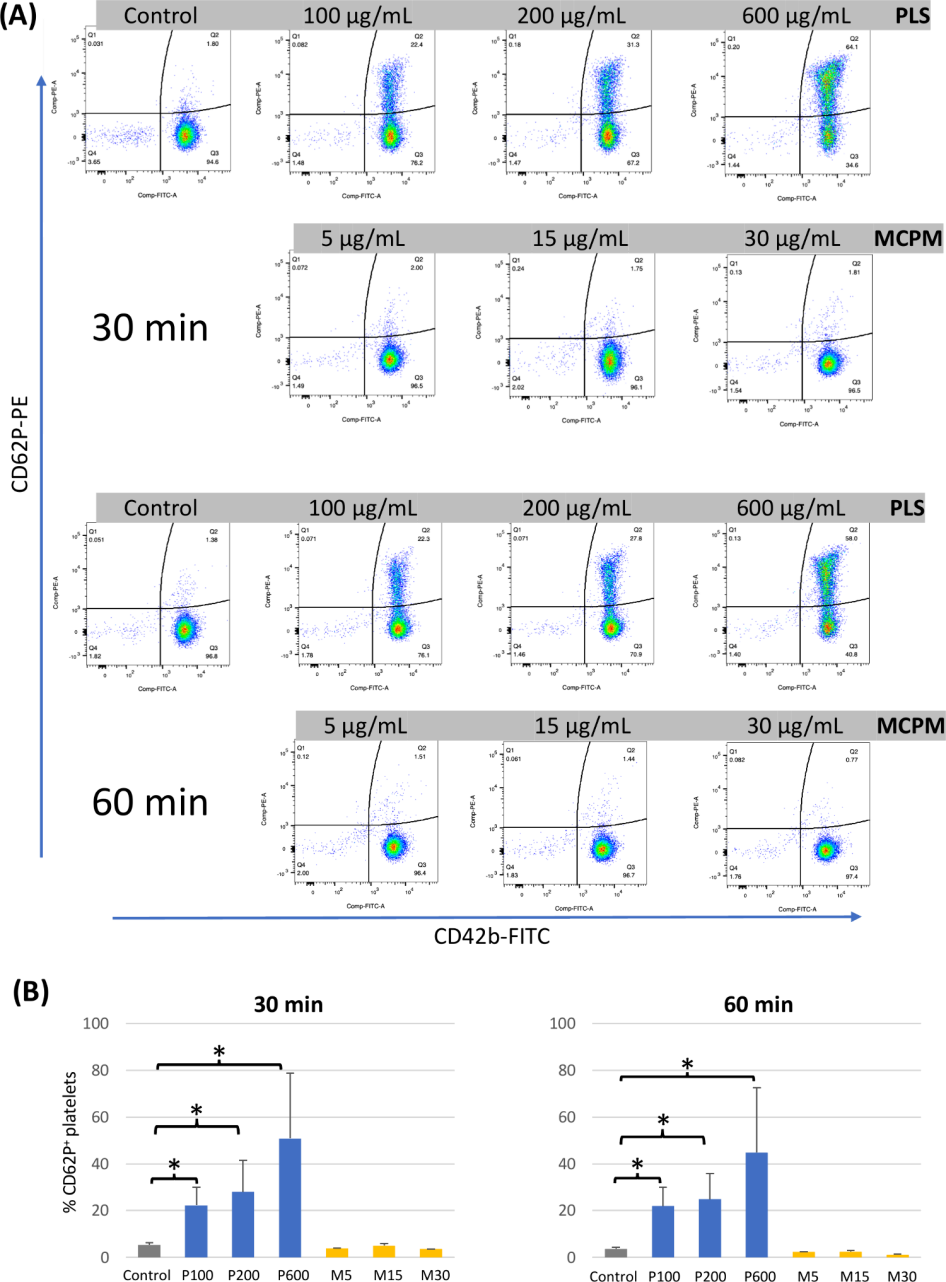
Effects of PLS and MCPM on platelet activation assessed by FCM. Human single-donor PRP (from 2 donors) were treated with PLS at 100, 200, and 600 μg/mL (P100, P200, and P600, respectively) and MCPM at 5, 15, and 30 μg/mL (M5, M15, and M30, respectively) for 30 and 60 min, and the cells were stained for CD42b and CD62P before FCM analysis. The gating strategy to measure the proportion of activated platelets (CD42b^+^CD62P^+^) is shown in (A), and a summary of % CD62P^+^ platelets individually induced by PLS and MCPM is shown in (B). Data are expressed as mean ± SD from two different single-donor PRP samples. *p* < 0.05.

Since only the 5P and 8M5P bone composites significantly activated platelets compared to the other groups, the roles of exogenously added PLS and MCPM in this activation were investigated. As shown in Figure 5A, unlike MCPM, PLS at all tested concentrations (100, 200, and 600 μg/mL) dramatically increased the proportion of CD62P^+^ platelets after both 30 and 60 min of exposure. Consistent with this observation, the analysis summarized in Figure 5B revealed a significant dose-dependent increase in the number of activated platelets by PLS. However, the proportions of activated platelets at 30 and 60 min appeared similar.

### Effect of Bone-Cement-Exposed Conditioned Plasma on *in Vitro* MSC-Mediated Wound Healing

**Figure 6:**
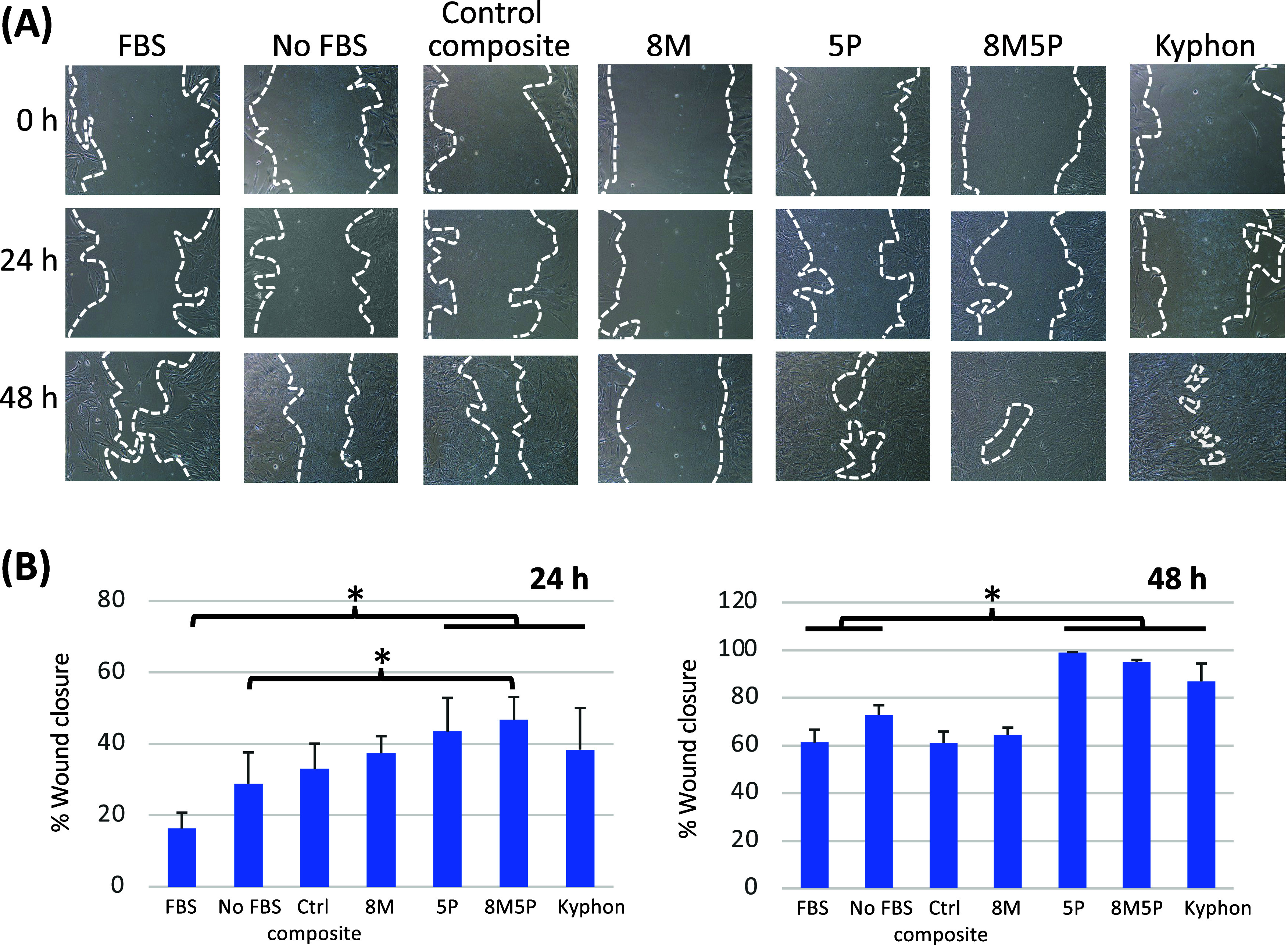
Effect of the bone-cement-exposed conditioned plasma on the *in vitro* MSC-mediated wound healing. Human PRP samples were incubated with the different bone cements for 1 h, and the cell-free conditioned plasma samples were used in the scratch-based wound healing assay. Representative phase-contrast microscopy images of wound closure after 24–48 h treatment with bone-cement-exposed conditioned plasma samples are depicted in (A), and analysis of the results is summarized in (B). Data are expressed as mean ± SD from three different single-donor PRP samples. *p* < 0.05.

The results of the scratch wound healing assay in Figure 6A showed that by following 24-h treatment with the conditioned plasma individually derived from 5P, 8M5P, and Kyphon bone cements, the initial and measurable wound closure was apparently observed. This marked reduction in wound gap compared to that of the control group was also evident after 48 h of treatment for the other groups. Notably, the 5P, 8M5P, and Kyphon groups exhibited the near-complete closure at the 48-h time point. Figure 6B depicts the analysis of wound closure percentage. Compared with that of the control group supplemented with FBS, the 5P, 8M5P, and Kyphon groups revealed a significant enhancement in wound closure, promoting it by approximately 2-fold. Notably, only the 8M5P group demonstrated a statistically significant acceleration in wound closure, compared to the control group lacking FBS. Furthermore, after 48 h of treatment, these three groups retained their ability to induce wound closure exceeding 85%, with the 5P and 8M5P groups demonstrating the most pronounced wound closure induction.

### Effects of Bone Cements on Cytokine Production

**Figure 7:**
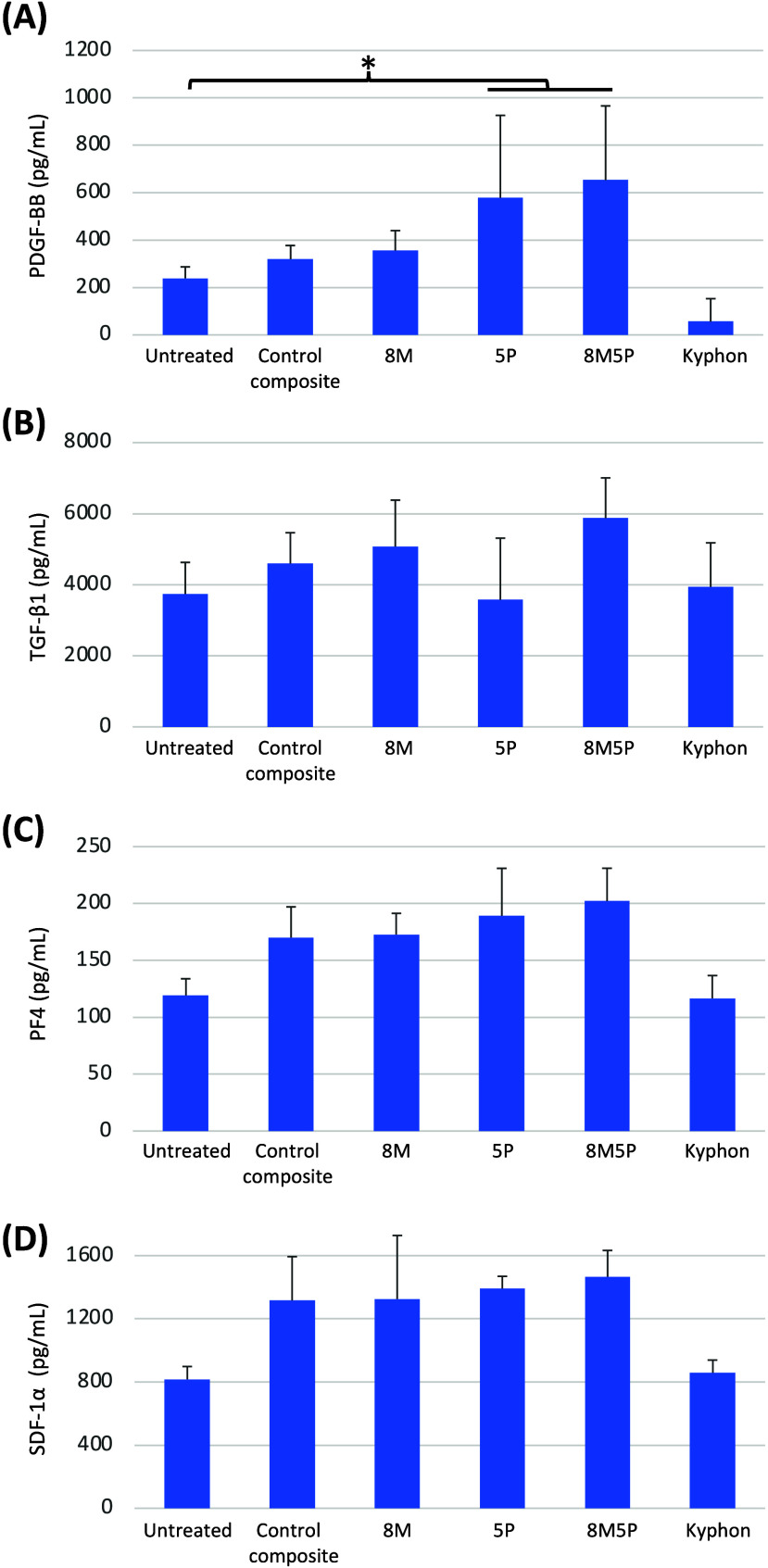
Effects of bone cements on the cytokine production assessed by ELISA. Human PRP was added to the individual bone cements at 37 °C for 60 min, and the levels of PDGF-BB, TGF-β1, PF-4, and SDF-1α were quantified using ELISA. Data are expressed as mean ± SD from three different single-donor PRP samples. *p* < 0.05.

To elucidate the mechanisms responsible for the wound closure induction observed with the bone-cement-exposed conditioned plasma, the levels of four key platelet-derived mediators implicated in MSC-mediated wound healing, i.e., PDGF-BB, TGF-β1, PF-4, and SDF-1α, were investigated. As depicted in Figure 7, among these four mediators, PDGF-BB exhibited a significant increase specifically following exposure to the 5P and 8M5P bone cements, with no such effect observed for Kyphon.

### Effect of PDGF-BB Neutralizing Antibody on Bone-Cement-Exposed Conditioned Media-Induced *in Vitro* MSC-Mediated Wound Healing

**Figure 8:**
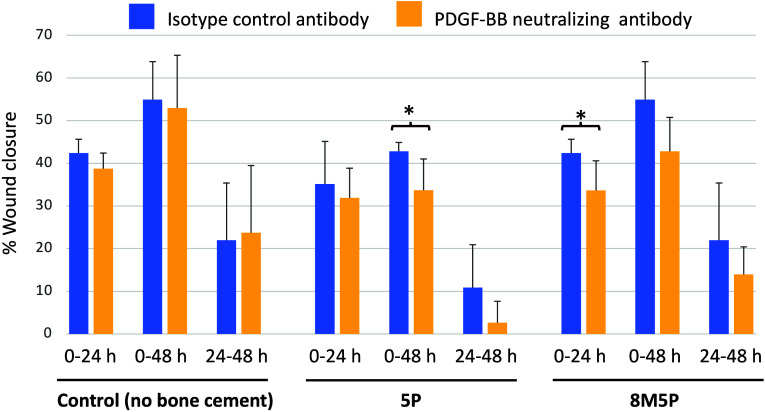
Effect of PDGF-BB neutralizing antibody on the bone-cement-exposed conditioned media-induced *in vitro* MSC-mediated wound healing. Human PRP was incubated with the different bone cements for 1 h, and the cell-free conditioned plasma samples were used in the scratch-based wound healing assay with either isotype control or PDGF-BB neutralizing antibodies for indicated times. Data are expressed as mean ± SD from three different single-donor PRP samples. *p* < 0.05.

The results in Figure 8 showed that PDGF-BB neutralizing antibody exhibited no inhibitory effect in the control group (no exposure to bone cement) compared to that of the isotype control antibody. Interestingly, the PDGF-BB neutralizing antibody appeared to reduce wound closure in the 5P and 8M5P groups at all time points analyzed. Statistically significant inhibition of wound closure by PDGF-BB neutralization was observed in the 5P and 8M5P groups at specific time points (i.e., 48 and 24 h for the 5P and 8M5P groups, respectively).

## Discussion

Platelets are essential for initiating bone fracture healing. This study examined the platelet response to novel UDMA-based bone cements containing 8% MCPM and/or 5% PLS and a commercial PMMA bone cement Kyphon. At 86% conversion, Kyphon, which contains monomethacrylate monomers, could not complete cross-linking; a significant 14% of unpolymerized toxic monomers might be released. Conversely, the UDMA-based bone cement developed in the present study could relatively minimize the potential for uncured toxic monomer leaching with its 75% conversion as only 50% conversion rate of dimethacrylate monomers theoretically enables complete cross-linking of all monomers when one methacrylate group of every monomeric molecule polymerized. Nevertheless, Kyphon and all the bone cement studied appeared to be nontoxic to platelets; platelet adhesion and aggregation were explicitly observed on all material surfaces (Figures 3A and 3B).

Platelet adhesion and activation depend on multiple surface characteristics of substrates, such as topography and wettability.^32^ All bone cements investigated, including Kyphon, exhibited rather smooth surfaces with Sa values ranging from 40 to 120 nm. Given the resting diameter of platelets at 3 μm, topographical features exceeding several microns in roughness are unlikely to directly influence platelet biological responses. Instead, they may function primarily as an expanded substrate for protein adsorption, thereby indirectly supporting platelet adhesion and activation. While the surface of Kyphon was somewhat hydrophobic, all UDMA-based bone composites studied were relatively more hydrophilic. It has been shown that hydrophobic polymers can suppress the activation and aggregation of platelets.^33^ It was hypothesized that platelets in contact with the hydrophobic polymeric surface require metabolic processes consuming ATP and involve dynamics of their membrane skeleton, which may reduce platelet activation.^34^

In a quiescent state, as found in the bloodstream, platelets exhibit a discoid morphology and minimal adhesive properties. Upon activation, a dramatic cytoskeletal reorganization occurs, transforming them into highly adhesive spheroid structures,^35^ as shown in this study following their exposure to the PLS-containing bone cements (Figure 3). This activation is mediated by a plethora of membrane receptors embedded within the platelet membrane. These receptors orchestrate a complex signaling cascade, culminating in morphological changes and the release of prestored and newly synthesized molecules to modulate the healing process.^36^ In the present study, exposure to 5P and 8M5P resulted in a notable augmentation of platelet adhesion, aggregation, and downstream signaling pathways. Specifically, significant increases in phosphorylated ERK, CD62P expression, and PDGF-BB secretion were observed. Furthermore, these two bone cements markedly enhanced the *in vitro* PDGF-BB-induced wound closure. The potential of 5P and 8M5P to stimulate the release of osteogenic mediators from platelets has yet to be fully elucidated. Future investigations are warranted to explore this aspect of their biological activity.

PLS has gained significant interest as a natural antimicrobial agent owing to its unique physicochemical properties.^37^ Compelling evidence from multiple studies has demonstrated PLS’s potent broad-spectrum antimicrobial activity against diverse pathogenic bacterial species.^38−41^ Notably, it has garnered approval from the FDA for incorporation as a food additive.^41,42^ Within the human body, PLS undergoes biodegradation into its constituent amino acid, l-lysine. This metabolic fate contributes to its safety profile, as l-lysine is a naturally occurring amino acid with minimal associated adverse effects.^41^ Prior investigations have demonstrated a direct correlation between the initial concentration of PLS (0.5, 1, and 2 wt %) within the composite filler and its subsequent release into an aqueous environment. Specifically, a 24-h incubation in water yielded PLS concentrations of 8, 25, and 93 ppm, respectively.^43^ It is noteworthy that the incorporation of PLS into the experimental composites presents a potential strategy to mitigate residual bacterial populations persisting after wound debridement and bone cement implantation at the fracture site. The present study presents the first-ever investigation of PLS regenerative potential within the UDMA-based bone cement formulations. PLS demonstrated the ability to effectively and rapidly induce platelet activation, both in its pure form and when incorporated within bone cement. This activation was observed to be concentration-dependent. Notably, the presence of MCPM, another active filler, did not appear to hinder the platelet activation induced by PLS. MCPM is known to rapidly dissolve from the bone cement surface, influencing the surrounding environment by promoting acidity and facilitating the formation of calcium phosphate precipitates. The combined use of MCPM and PLS within the bone cement formulations remains advantageous due to its potential benefits for enhanced bone formation, antimicrobial activity, and overall regenerative potential of the implanted material.

While Akt signaling is considered the primary intracellular pathway governing platelet activation, potentially impacting thrombosis,^44^ this study observed a decrease in Akt activation with all tested bone cements. However, CD62P, a crucial platelet surface marker associated with functional consequences, showed a significant increase upon exposure to the PLS-containing formulations (i.e., 5P and 8M5P) and exogenously added PLS for up to 30–60 min (Figures 4 and 5, respectively). These findings suggest the involvement of Akt-independent signaling pathways in the platelet activation induced by the PLS-containing bone cements investigated here. In addition to Akt signaling, within platelets two mitogen-activated protein kinases (MAPKs), i.e., ERK and p38 MAPK, have been implicated in signaling pathways.^17−20^ Studies have also demonstrated that the ERK signaling cascade is activated in platelets by exposure to thrombin or collagen, potentially involving the intermediary kinases MEK1/2 and protein kinase C (PKC).^45,46^ It is possible that these MAPKs may also contribute to platelet activation by the 5P and 8M5P formulations. Further investigation is warranted to confirm this hypothesis.

The precise mechanism through which PLS interact with platelets remains unclear. However, it is plausible that the positively charged PLS molecule binds to GPVI, a member of the immunoglobulin (Ig) receptor superfamily expressed on platelets.^47^ GPVI is activated by a diverse range of endogenous and exogenous ligands, including positively charged ligands such as histones.^48^ GPVI has several charge areas in its two Ig domains and contains a highly negatively charged stalk that is highly O-glycosylated and charged due to sialylation.^49,50^ Future investigations are required to validate this hypothesis.

PDGF-BB emerges as a frontrunner for therapeutic intervention in wound healing due to its well-established safety profile and multifaceted influence on cellular processes critical for tissue regeneration, being regarded as a promising therapeutic candidate in wound healing.^51,52^ A study by Jian et al.^53^ demonstrates its ability to orchestrate both collagen deposition and angiogenesis, two processes that represent critical cornerstones within the wound-healing cascade. Our findings from the scratch-based wound healing and PDGF-BB neutralization assays suggested that PDGF-BB, at least partially, mediates the *in vitro* enhancement of wound healing observed with the 5P and 8M5P formulations. However, the present study did not explore the mechanism by which these specific bone cement formulations upregulate PDGF-BB. Previous research has identified several signaling pathways in platelets, including Ras/Raf/MEK/ERK system, that may contribute to this process.^54^ Furthermore, it would be valuable to investigate whether PDGF-BB, induced by the bone cements described in this study, also plays a role in other aspects of *in vivo* wound healing, as previously reported.

Prior studies have demonstrated that incorporating platelet-derived mediators, such as those found in platelet gels and platelet-rich plasma (PRP), can augment the regenerative capacity of polymethylmethacrylate (PMMA) bone cements. For instance, platelet gel, a rich source of growth factors, cytokines, and molecules essential for bone formation and remodeling, has been shown to enhance the bone regenerative properties of PMMA bone cement *in vivo* compared to PMMA alone.^55^ Including PRP in calcium sulfate hemihydrate bone cement has been shown to enhance its biological activity.^56^ The developed PLS-containing bone cements can activate endogenous platelets, producing cytokines and growth factors without exogenous platelet products. This approach offers several advantages over incorporating autologous platelet products, which can potentially influence the properties of bone cements and may require extensive optimization. Furthermore, the withdrawal and processing of blood for autologous platelet products can prolong the surgical procedure due to additional clinical and laboratory steps. While PLS has been approved by FDA for use as a food additive, a thorough investigation of the safety of the PLS-containing UDMA-based bone cements is necessary before clinical application.

## Conclusion

Addition of MCPM and/or PLS into experimental UDMA-based bone cements had negligible effect upon monomer conversion or hydrophilicity. PLS-containing UDMA-based bone cements promoted platelet activation, as evidenced by elevated CD62P expression. PDGF-BB secretion was significantly elevated by PLS-containing UDMA-based bone cements, contributing to *in vitro* MSC-mediated wound closure. These findings suggest that PLS plays a key role in enhanced platelet activation and wound closure by UDMA-based bone cements *in vitro*. This warrants further *in vivo* animal studies.

## Supporting Information Available

Components of the liquid phases in the initiator and activator pastes (PDF)

## Notes

This study was performed in accordance with the principles of the Declaration of Helsinki. The Ethics Review Sub-Committee for Research Involving Human Research Subjects of Thammasat University No. 3 (COA No. 068/2564), the Institutional Biosafety Committee of Thammasat University (057/2564), and the Institutional Review Board of the Faculty of Medicine Siriraj Hospital (COA. No. 733/2557 (EC1), Si101/2015) granted approval.
