# Supplementary material for: Platelet Responses to Urethane Dimethacrylate-Based Bone Cements Containing Monocalcium Phosphate/ε-Polylysine: Role of ε-Polylysine in In Vitro Wound Healing Induced by Platelet-Derived Growth Factor-BB
Source: ACS Mater Au. 2025 Jan 3;5(2):339–52. doi: 10.1021/acsmaterialsau.4c00143 (PMC11907285; doi:10.1021/acsmaterialsau.4c00143)
Supplement: Supplementary file 1 — mg4c00143_si_001.pdf [file mg4c00143_si_001.pdf]

Supporting Information

Components of the liquid phases in the initiator and activator pastes

Platelet responses to urethane dimethacrylate-based bone cements containing monocalcium phosphate and ε-polylysine: Role of ε-polylysine in *in vitro* wound healing induced by platelet-derived growth factor-BB

Phatchanat Klaihmon[1], Piyarat Sungkhaphan[2], Boonlom Thavornyutikarn[2], Setthawut Kitpakornsanti[3], Praphasri Septham[1], Anne Young[4], Chanchao Lorthongpanich[1,5], Wanida Janvikul[2*] and Weerachai Singhatanadgit[3*]

[1]Siriraj Center of Excellence for Stem Cell Research, Faculty of Medicine Siriraj Hospital, Mahidol University, Bangkok 10700, Thailand

[2]National Metal and Materials Technology Center, National Science and Technology Development Agency, Pathum-thani 12120, Thailand

[3]Faculty of Dentistry and Research Unit in Mineralized Tissue Reconstruction, Thammasat University (Rangsit Campus), Pathum-thani 12121, Thailand

[4]Division of Biomaterials & Tissue Engineering, UCL Eastman Dental Institute, London NW3 2PF, U.K.

[5]Blood Products and Cellular Immunotherapy Research Group, Faculty of Medicine Siriraj Hospital, Mahidol University, Bangkok 10700, Thailand

*Authors for correspondence

Table S1. Components of the liquid phases in the initiator and activator pastes.

Upon mixing each composite formulation, BPO and NTGGMA concentrations will become 1.5 and 1 wt%, respectively.

Liquid phases	UDMA	PPGDMA	HEMA	BPO	NTGGMA
	(wt% of monomers)			(wt% of liquid)	
Initiator phase	64	32	2.5	3	0
Activator phase	64	32	2.5	0	2
